# Fabrication of superhydrophobic and ice-repellent surfaces on pure aluminium using single and multiscaled periodic textures

**DOI:** 10.1038/s41598-019-49615-x

**Published:** 2019-09-26

**Authors:** Stephan Milles, Marcos Soldera, Bogdan Voisiat, Andrés F. Lasagni

**Affiliations:** 1 Technische Universität Dresden, Institut für Fertigungstechnik, George-Bähr-Str. 3c, 01069 Dresden, Germany; 2 PROBIEN-CONICET, Dto. de Electrotecnia, Universidad Nacional del Comahue, 8300 Neuquén, Argentina; 3 Fraunhofer-Institut für Werkstoff- und Strahltechnik (IWS), Winterbergstr. 28, 01277 Dresden, Germany

**Keywords:** Aerospace engineering, Mechanical engineering

## Abstract

Fabricating aluminium surfaces with superhydrophobic and ice-repellent properties present nowadays a challenging task. In this work, multifunctional structures are manufactured by direct laser writing and direct laser interference patterning methods using pulsed infrared laser radiation (1064 nm). Different periodic patterns with feature sizes ranging from 7.0 to 50.0 µm are produced. In addition, hierarchical textures are produced combining both mentioned laser based methods. Water contact angle tests at room temperature showed that all produced patterns reached the superhydrophobic state after 13 to 16 days. In addition, these experiments were repeated at substrate temperatures from −30 °C to 80 °C allowing to determine three wettability behaviours as a function of the temperature. The patterned surfaces also showed ice-repellent properties characterized by a near three-fold increase in the droplets freezing times compared to the untreated samples. Using finite element simulations, it was found that the main reason behind the ice-prevention is the change in the droplet geometrical shape due to the hydrophobic nature of the treated surfaces. Finally, dynamic tests of droplets imping the treated aluminium surfaces cooled down to −20 °C revealed that only on the hierarchically patterned surface, the droplets were able to bounce off the substrate.

## Introduction

Currently, modification of aluminium surfaces to obtain superhydrophobic and ice-repellent properties presents a challenging task. These properties have shown recently an increased importance due to the many different applications where aluminium is used^1–3^. In general, superhydrophobic and anti-icing properties can be achieved either by changing the surface chemistry, by controlling the surface topography or by modifying both simultaneously^4^. These extreme wetting behaviour can be technically used for liquid transportation, self-cleaning applications, anti-fouling or for corrosion prevention^5–7^. Furthermore, non-sticking surfaces also can lead to a reduction of snow-accumulation^8–10^. Very well-known natural examples as the lotus leaf (nelumbo nucifera) represent a perfect template for the mechanism of water repellent properties. In this frame, many attempts have been performed to achieve this wetting characteristic^11^. For example, it was reported that the use of a silane coating or chemical etching in various acids can lead to superhydrophobic surfaces^12^. Besides of chemical treatments, micro- and/or nanostructured surfaces have also been used to control wettability properties. For instance, using regular and repetitive patterns it was possible to produce surfaces presenting air-cushion like behaviour resulting in lower adhesion and thus leading water droplets to easy roll at the materials’ surface (rolling-off phenomena)^13^. In particular, triangular-like patterns were fabricated on aluminium using photolithography methods, obtaining both hydrophobic and ice-retarding characteristics^14^. In addition, it was also reported that micro/nano-rough aluminium surfaces showing hydrophobic properties can reduce the anti-icing behaviour and thus forming ice even faster^15^. Recently. Recently, also laser processed aluminium samples in combination with UV irradiation as well as the application of a chemical coating lead to longstanding icephobic surfaces^16^.

Applications requiring superhydrophobic properties on aluminium or aluminium based alloys are of significant importance in several industrial sectors. For instance in the automotive sector, hydrophobic surfaces protect cars from moisture, which results in less corrosion and a prolonged shelf life^17^. Additionally to the corrosion resistance, it was reported a superhydrophobic surface that shows an increased wear resistance against NaCl particles, which can be applied for the marine industry^18^. Ice accretion on any kind of material leads to different problems and safety risks especially in cold regions on the world^4^. For example, ice formation on offshore platforms represents an important safety issue, since it can be formed on complex constructions^19^. In the aviation industry, ice-repellent surfaces can reduce the in-flight icing phenomena and thus decreasing energy losses^20^.

In addition to the methods mentioned above, it has been also shown that treatments using pulsed laser sources (e.g. nanoseconds pulses) are capable to create superhydrophobic as well as anti-corroding surfaces on aluminium-magnesium alloys^21^. This laser treatment also permitted to obtain superhydrophobic properties on polymer films^22^ and titanium alloys, among other materials^23^.

One of the typical methods for manufacturing hydrophobic surfaces using laser sources is direct laser writing (DLW). DLW has been used to treat aluminium surface producing square-like patterns with distances ranging between 10 and 35 µm. These surfaces have shown water contact angles even over 150°^24,25^. In DLW, the resolution is generally limited to 10–20 µm (depending on the focusing optics, beam diameter and laser wavelength) and typically the laser beam can be moved over the material surface with a scanning speed up to 15 m/s^23,25–29^. An alternative and innovative technique for producing periodic structures with significantly smaller features is Direct Laser Interference Patterning (DLIP)^30,31^. In DLIP, two or more coherent laser beams are overlapped on the material surface which interfere and thus create a periodic variation of the laser energy. Depending on the number of interfering laser beams, the laser wavelength, the beam polarization, the angle of incidence and the geometrical arrangement of the beams, very different interference patterns can be obtained, even with a resolution in the sub-micrometer range^32,33^. The spatial period of the interference pattern Λ is mainly controlled by the intercepting angle between the individual laser beams. In the case of a two-beam configuration, a line-like geometry can be obtained and its spatial period Λ can be calculated using Eq. 1:

1 $$\varLambda =\frac{\lambda }{2\,\sin (\beta /2)}$$

where *λ* is the laser wavelength and *β* the angle between the two interfering laser beams. Compared to other surface structuring technologies, any additional process is required and the patterns can be produced even using a single laser pulse, depending on the morphological characteristic of the desired pattern^34^. The DLIP technology has been used for producing functional surfaces on various materials and application fields. For instance, the efficiency of organic solar cells could be increased up to 35% (relative) using triangular-like arranged DLIP structures^35^. Also, periodic magnetic structures in Co/Pd thin films were produced and seamless sleeves for hot embossing processes on polymer films were manufactured^36,37^. Additionally, for medical purposes, DLIP produced structures were used to regulate the cell growth mechanism (e.g. for neuronal cells) and to generate antibacterial properties^38,39^ or for friction reduction in deep drawing processes^40^. Furthermore, the wettability of stainless steel surfaces could be controlled using this method as well^41,42^.

Although this important amount of research, any investigation concerning the application of DLIP, DLW as well as both combined methods has been performed for obtaining superhydrophobic and ice-repellent surfaces up to now. In this context, this work outlines the manufacturing process of multifunctional structures on aluminium using DLW and DLIP methods. For the DLW process, structures with a triangular-like arrangement are produced with of 50 µm separation distance, while pillar-like structures with a spatial period of 7.0 µm are produced using DLIP. In addition, multiple-scale patterns are also fabricated combining both methods. Contact angle measurements of all laser textured surfaces are performed by varying the temperature from −20 °C to 80 °C. To investigate the ice-repellent properties, the water droplet freezing time is recorded and dynamic analyses are performed to elucidate the influence of the different topographical feature sizes on the anti-icing behaviour. Finally, the experiments are supported by finite element method simulations.

## Results and Discussion

In order to examine the influence of different topographical features with specific sizes on both wettability and ice-repellent properties, three different types of textured surfaces were manufactured on pure aluminium substrates (Al2024). The parameters for producing these textures were selected according to a previous research work, which described the influence of different topographical parameters (e.g. structure height, hatch-distance, spatial period and pattern geometry) on the wettability of pure aluminium Al2024 at ambient temperature^43^.

In the following sections, these three different types of morphologies will be introduced, including (i) triangular-like structures produced using DLW; (ii) pillar-like structures fabricated using DLIP; and (iii) multiscaled textures resulting from the combination of DLW and DLIP. After describing the fabrication procedures and the morphological characteristic of the produced structures, both the wettability and icing behaviour are evaluated.

### Fabrication of periodic structures using DLW and DLIP methods

For producing the triangular-like structures, direct laser writing process was employed using infrared laser radiation (1064 nm wavelength) with nanosecond laser pulses (14 ns pulse duration). The parameters to process the samples were fixed to a laser fluence (for each pulse) of 1.06 J/cm² and a scan speed of 250 mm/s, while the hatch distance was 50 µm (see experimental section for additional information). Three separate line-like structures were manufactured with a rotation of 60° to each other, resulting in the triangular-like geometry. As mentioned before, these parameters have been taken from a previous work^43^.

**Figure 1:**
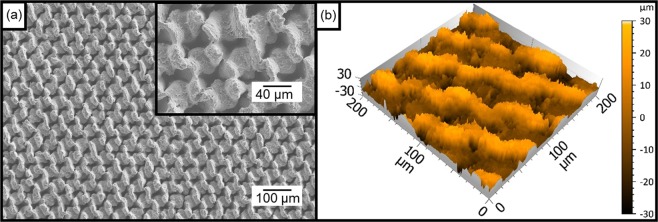
(**a**) SEM and (**b**) confocal images of the DLW treated aluminium surface using a hatch distance HD of 50 µm with a laser fluence of 1.06 J/cm², a scanning speed of 250 mm/s at a repetition rate of 30 kHz. 10 scan repetitions were used for each trench (at 0°, 60° and 120°). The pulse duration of the laser was 14 ns and the used wavelength was 1064 nm.

In Fig. 1a, a scanning electron microscope (SEM) image of the produced triangular structure is shown exemplarily. Since the spot size of the laser radiation at the focal position had a diameter of approximately 70 µm, which is larger than the hatch distance (HD = 50 µm), the obtained surface topography on the Al substrate shows a large amount of molten material with a close meshed characteristic. Furthermore, the lines produced during the last (3^rd^) scanning step are dominant. In addition, since all trenches are merged, no untreated areas in between are visible, which means that the total area of the substrate was molten by the laser process which is typical when ns-pulses are used^44,45^. The three-dimensional topography (confocal microscope image) of this pattern is also shown in Fig. 1b. As it can be observed, the resulting structure depth was 36.8 µm.

**Figure 2:**
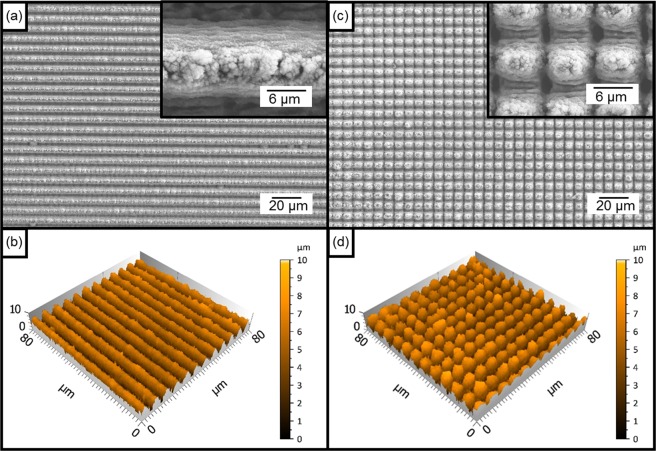
(**a**) SEM and (**b**) confocal images of the line-like DLIP and (**c**) SEM image and (**d**) confocal image of the pillar-like DLIP treated aluminium surface using a spatial period of 7.0 µm, a laser fluence of 1.93 J/cm² and a pulse overlap of 99%. The pulse duration of the laser was 10 ps and the wavelength was 1064 nm.

For producing surface textures with significantly smaller feature sizes, DLIP was used. The patterns consist of pillar-like structures with a spatial period of 7.0 µm. This geometry can be obtained using a two-beam configuration producing first a line-like geometry which turns into the pillar geometry when the sample is re-irradiated after rotating the substrate by 90° (see “Materials and Methods” section). Figure 2a,c show representative SEM images of the line-like and pillar-like patterns obtained after the first and second irradiation steps, respectively. In addition to the 7.0 µm features, smaller structures are also visible which have been described as Laser Induced Periodic Surfaces Structures (LIPSS). These features have a spatial period of ~570 nm, which is in the order of magnitude of half of the used wavelength and are typical for materials that are irradiated with laser pulses with durations in the ps range^44–46^ (see insets in Fig. 2). Furthermore, the observed LIPSS are perpendicular to the used laser polarization direction, which is typical for the so called low spatial frequency LIPSS (LSFL). The topography of both surfaces was also characterized by using confocal microscopy as it is shown in Fig. 2b,d. Thereof the resulting structure depth was 4.5 µm for the line-like structure and 5.8 µm for the pillar-like structure.

**Figure 3:**
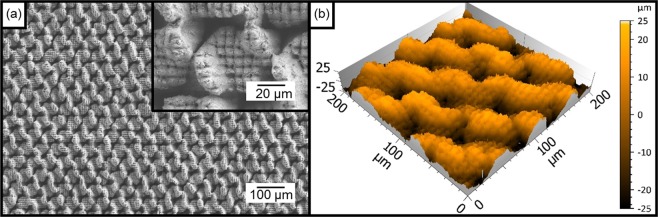
SEM (**a**) and confocal images (**b**) resulting from the combination of DLW and DLIP processing on aluminium. DLW used a hatch distance HD of 50 µm, a laser fluence of 1.06 J/cm² and a scanning speed of 250 mm/s. 10 scan repetitions were used for each trench (at 0°, 60° and 120°). The pulse duration of the laser was 14 ns. The DLIP processing used a spatial period of 7.0 µm, a laser fluence of 1.93 J/cm², a pulse overlap of 99% and a pulse duration 10 ps. The wavelength was 1064 nm for both structuring methods.

Finally, both above described methods were used for fabricating multiscale hierarchical patterns. The used processing parameters for each individual method were the same as for the single-scale cases. To preserve the morphology of the small features, the Al substrates were firstly treated with DLW (producing the 50 µm features) and later using DLIP (using 7.0 µm of spatial period). SEM and confocal microscopy images showing the resulting structure are depicted in Fig. 3. As it can be observed, the DLIP structure is visible on top as well as on the bottom of the DLW features. It has to be mentioned, that during the DLIP process neither the substrate nor the interfering beams are vertically translated (perpendicularly to the sample surface) since the size of the volume where the laser beams interfere is much larger than the structure height of the DLW features (see section “Materials and Methods”). From Fig. 3b, the measured structure depth of the pillar features was ~4.2 µm while for the DLW it was 33.8 µm.

### Evolution of the wetting properties

**Figure 4:**
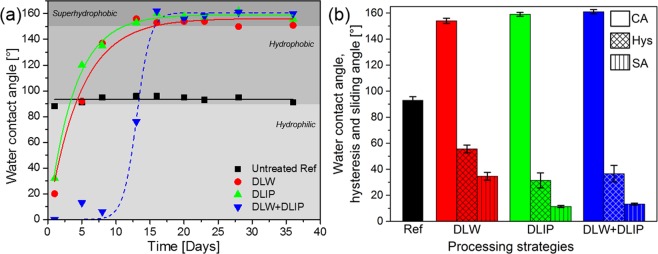
Static water contact angle measurements (8 µl deionized water droplets) on the untreated reference and substrates treated using DLW, DLIP, DLW + DLIP methods. The solid lines for the DLW (green line) and DLIP (red line) processed surfaces are the fits calculated using Eq. (2). The blue dashed line representing the wetting behaviour of the substrates treated with DLW + DLIP is a guide to the eye only (**a**) and the corresponding contact angle (CA), hysteresis (Hys) and sliding angles (SA) (**b**).

In order to examine the wetting properties of the fabricated structures, static water contact angle (WCA) measurements were carried out with deionized water droplets of 8 µl. The measurements were started on the second day after the laser process and were continued over a 36 days period. The WCAs of the untreated reference surface as well as DLW, DLIP and DLW + DLIP patterned surfaces were measured. The obtained results are shown in Fig. 4a.

As it can be seen, the WCA of the untreated reference surface (Fig. 4a, black line) stayed almost constant with an average value of 93° over the studied time period (the WCA oscillated between 88° and 96°). This value is around the boundary of transition from hydrophilic to the hydrophobic state, corresponding to a contact angle of 90°^47^. On the other hand, the samples treated with either DLW or DLIP methods exhibit an increase of the contact angle over time, which can be described using an exponential growth regression fit proposed by Kietzig *et al*.^48^

2 $$\theta ={\theta }_{max}\cdot (1-{e}^{(-at)})$$

where *θ*_*max*_ is a maximum contact angle and *a* is a time constant. By fitting the experimental data with this equation, the values shown in Table 1 were obtained.

**Table 1:** Maximum contact angle (θ_max_) and time constant (*a*) corresponding to the exponential growth regression fits for the DLW and DLIP treated samples.

Structure	*θ*_*max*_	*a* (days^−1^)
DLW	156° ± 4°	0.21 ± 0.025
DLIP	159° ± 2°	0.26 ± 0.014

The increase of the WCA has been explained by the change of the surface chemistry over time. Due to the thermal influence of the laser process, firstly an aluminium oxide layer (Al_2_O_3_) if formed with a large number of polar sites composed of unsaturated aluminium and oxygen atoms leading to a hydrophilic condition^6,18,24,49,50^. This behaviour has been observed for all laser treated samples on the first day of measurements, as it can be seen in Fig. 4a. Later, an increase in the carbon content at the Al surface from the ambient air occurs, increasing the number of non-polar sites and thus raising the WCA^48^. Such evolution of WCA on laser treated aluminium has already been discussed in detail in a previous work^43^.

After 8 days, the DLW and DLIP processed samples showed similar wetting properties and turned from the hydrophilic into the hydrophobic state (WCA between 90° and 150°^47^) with 137° and 135° for the samples treated with the DLW and DLIP methods, respectively. Finally, after 16 days all the laser treated samples turned into the superhydrophobic state (defined with a static WCA higher than 150°^47^) resulting in WCAs of 153° for DLW and 160° for DLIP. The reached superhydrophobic state did not change until the end of the performed measurements (36 days). The observed superhydrophobic behaviour was determined to be controlled by the obtained microstructure, providing air cushions between the water droplet and the material leading to the Cassie-Baxter state^13,43^.

In the case of the Al samples treated with both methods (DLW + DLIP, see blue data points in Fig. 4a), a slightly different behaviour was observed. In this case, direct after the laser treatment the samples showed also a hydrophilic behaviour, but the WCA did not increase during the first 8–10 days. After that, the WCA strongly increased reaching also the superhydrophobic state (WCA > 150°), similarly to the other two studied cases. Such shift of the WCA growth for the DLW + DLIP treated samples might be caused by chemical changes by the second laser treatment (DLIP) on top of the previous DLW features. XPS measurements for this hierarchical DLW + DLIP structure were done 2 and 20 days after the laser process. The chemical composition for the elements aluminium, carbon and oxygen were 16.5 at%, 34.53 at%, and 48.97 at%, respectively after 2 days, and 17.14 at%, 34.53 at%, and 48.33 at%, respectively after 20 days. As it can be seen, any significant change in the surface chemical composition was observed, and thus the chemical composition alone cannot explain the observed shift in WCA evolution. Moreover, the reported values are very similar to the chemical composition of Al substrates treated only using DLIP, which means that the interference treatment partially (or totally) ablates the material that has been modified by the DLWprocess^43^. A more detailed study has to be performed in the future to address this unusual behaviour.

Additionally to the static contact angle measurements, also the contact angle hysteresis, defined as the difference between the advancing and the receding contact angle during the sliding of a droplet on a tilted sample, was measured after 95 days (Fig. 4b)^51^. Compared to the previous measurements done after 36 days, no significant difference was observed in the static contact angle proving the long term superhydrophobicity. As it can be seen, in the DLW treated substrates the contact angle hysteresis (55°) and the sliding angle (34°) are significantly higher than the values measured for the DLIP (31° and 11°, respectively) and DLW + DLIP (36° and 13°, respectively) treated samples. These differences could indicate, that in case of the DLW samples, the wetting characteristic is controlled by the Wenzel regime, while for both the DLIP and DLW + DLIP samples, the Cassie-Baxter wetting state dominates^51,52^.

### Temperature influence on the wetting behaviour

**Figure 5:**
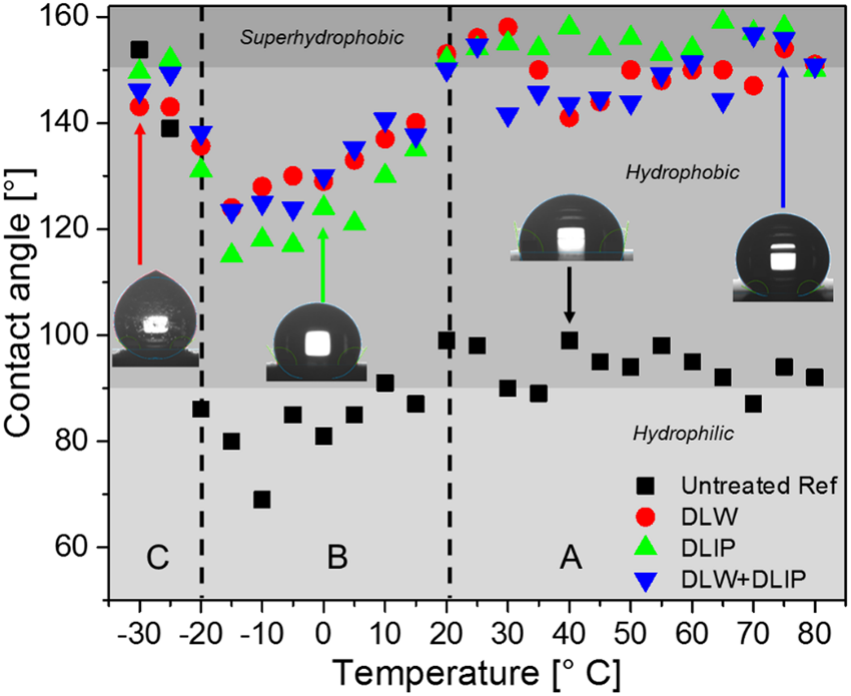
Contact angle measurements performed on the untreated reference as well as DLW, DLIP, DLW + DLIP irradiated samples using 8 µl of deionized water. The inserted images of the droplets provide an impression of the measured angles depending on temperature.

After examining the wetting properties of the laser structured surfaces for 36 days at a constant room temperature of 20 °C, the wetting behaviour of 8 µl droplets placed on the samples held at temperatures ranging from −30 °C to 80 °C were studied (see “Materials and Methods” section). According to the obtained results shown in Fig. 5, three different regimes can be identified (A, B and C). In regime A, with temperatures ranging from 20 °C to 80 °C, the WCA of all studied surfaces did not show a significant change depending on the substrate temperature. For instance, the WCA of the untreated sample oscillated around 87° and 99°. Like the untreated reference, the laser-structured samples also showed almost constant WCAs oscillating around 150°.

In regime B, defined for temperatures between −20 °C and 20 °C, it can be observed a linear dependence of the WCA with decreasing temperatures for all examined samples. This behaviour has been attributed to condensation of moisture from the environment on the materials’ surface at temperatures slightly above 0 °C, which leads to the so-called Cassie-Wenzel state resulting in lower contact angles^53^. As the temperature decreases further, frost on the sample surface builds up due to ice nucleation from the vapour phase at temperatures below 0 °C leading to the loss of superhydrophobicity^53–55^. These two physical effects lead to the partial spreading of the droplet and thus to a decreased WCA^4,10,56–58^.

The regime C covers the temperature range between −30 °C and −20 °C. In this case, due to the significant lower sample temperature, the droplets began freezing at the very first contact with the surface. Therefore, the droplets cannot completely spread as they would do at higher temperatures. This behaviour is more pronounced at temperatures below −25 °C, where even the untreated reference showed high contact angles (between 139° and 154°). The same behaviour was also observed for the laser treated samples. An indicator for the completed icing process is the tip forming on top of the droplet (see insert in Fig. 5 for regime C). In the next section, the icing dynamics of these structures is characterized to gain a deeper insight into the ice-repellent properties.

### Icing examinations of micro-structured surfaces

**Figure 6:**
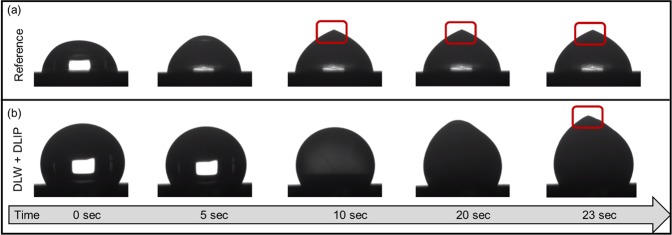
Different snapshots of a droplet solidification process on an untreated reference sample (**a**) compared to a DLW + DLIP structure recorded over a time period of 23 seconds (**b**). The red boxes indicate the formed tip of the frozen droplet.

In order to characterise the ice-repellence properties of the studied surfaces, the freezing dynamics of 8 µl water droplets placed onto these samples were recorded. For instance, Fig. 6a shows the freezing process of a single droplet at −20 °C over a time period of 23 s on a reference and on a DLW + DLIP processed sample (Fig. 6b). The recording time started upon the first contact between the droplet and the surface and ended as soon as the tip formed on top of the droplet resulting the complete solidification (red boxes in Fig. 6 point to the formed tip).

**Figure 7:**
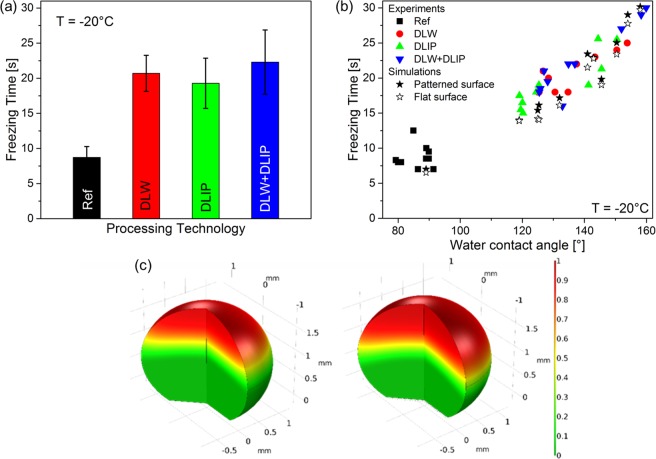
Average freezing time of 8 µl droplets on an untreated reference, DLW, DLIP, DLW + DLIP structures at −20 °C. (**a**) Experimental and simulated freezing time as a function of the water contact angle at −20 °C of patterned and flat surfaces. (**b**) Simulation of the phase transition from a liquid to a solid phase of the 8 µl droplet with a WCA of 141° on a flat (left) and a patterned (right) surface after 15 s (**c**).

The bar diagram in Fig. 7a shows an overview of the average freezing times of ten droplets placed onto each studied surface. The error bars represent the respective standard deviation. On the untreated reference, the droplets froze the fastest after an average time of 8.7 s. Remarkably, droplets on the DLW + DLIP structure froze on average after 22.3 s, representing a near three-fold increase compared to the reference. The results presented in Fig. 7a correspond to 10 icing and deicing cycles for each sample. Neither contact with external components nor mechanical abrasion took place on the surface during the tests. Thus, it can be assumed that the microstructures are stable with respect to the conducted icing test. However, further experiments regarding the mechanical stability of the structure have to be performed in the future.

Upon plotting the freezing time as a function of WCA of every single measured droplet (Fig. 7b), a positive correlation regardless of the studied topography was observed, as it was already reported elsewhere^59,60^. There are two main factors explaining this effect. On the one hand, as the contact angle increases, less effective droplet area is in contact with the cold surface reducing the total heat transfer between droplet and cold plate and slowing the solidification. On the other hand, for a constant droplet volume, an increase in the contact angle implies an increase in the droplet height, which in turn increases the droplet internal thermal resistance and delays the freezing process^59,61^. A third factor possibly influencing the delayed freezing time could be the increased thermal resistance between sample and droplet caused by the trapped air between the pattern cavities and the droplet. To evaluate this hypothesis, finite element method simulations were carried fowling the procedure described in the “Materials and Methods” section. The solid stars in Fig. 7b correspond to the simulation results assuming a structured surface with a spatial period of 50 µm and a structure height of 35 µm (comparable to the DLW structure geometries), while the open stars shows the simulated freezing time considering that the surface is perfectly flat, i.e. the whole droplet bottom surface is in contact with the cold aluminium surface. Figure 7c shows the simulated phase transition from the liquid (“1”, red) to the solid phase (“0”, green) of a droplet with a contact angle of 141° on a flat (left hand side) and on a patterned surface (right hand side) after 15 s. It can be seen that the water-ice interface (yellow) in case of the patterned surface lies below, i.e. is delayed compared to the flat surface. Recalling Fig. 7b, even though for WCAs between 120° and 125° the simulation and experimental results diverge up to 20%. For WCA over 137°, this difference is reduced to less than 10%. It is worth to mention, that since the used model does not take into account the droplet volume expansion upon water solidification, the increase in the final droplet height is disregarded. As a consequence, it is anticipated that the simulated freezing time could be lower than the experiment results, as it is in the case, for instance, for droplets with WCA < 125°. Despite some fluctuations, probably caused by the deviation of the droplet initial volume from the nominal volume of 8 µl, the general trend observed for the experimental data is satisfactorily followed by the numerical model.

As expected, the simulated freezing time is in all cases higher for the patterned surface than for the corresponding flat plate due to the increased thermal resistance generated by the trapped air. Interestingly, the average difference in the simulated freezing time between these surfaces is only 6%. Moreover, simulations with different patterns, namely spatial periods of 7 µm and 25 µm and structure heights of 5 µm and 15 µm, respectively, revealed that the average freezing time difference between patterned and flat surfaces is even lower than 6%. Therefore, it can be concluded that the trapped air in the cavities does not act as an effective thermal insulator and is not a main factor influencing the freezing time. The experimental results also confirm this conclusion since there is no clear correlation between the employed patterning technology or produced topography and freezing time as shown in Fig. 7b.

### Dynamic droplet impingement behaviour

To further characterise the ice-repellent properties on the studied surfaces, dynamic droplets impingement tests were carried out to complement the static water contact angle measurements. In the following experiments, droplets with a volume of 13 µl were dropped from a height of 20 mm onto the samples cooled down to −20 °C at 12% ambient air humidity. The process was recorded with a camera at a frame rate of 200 fps.

**Figure 8:**
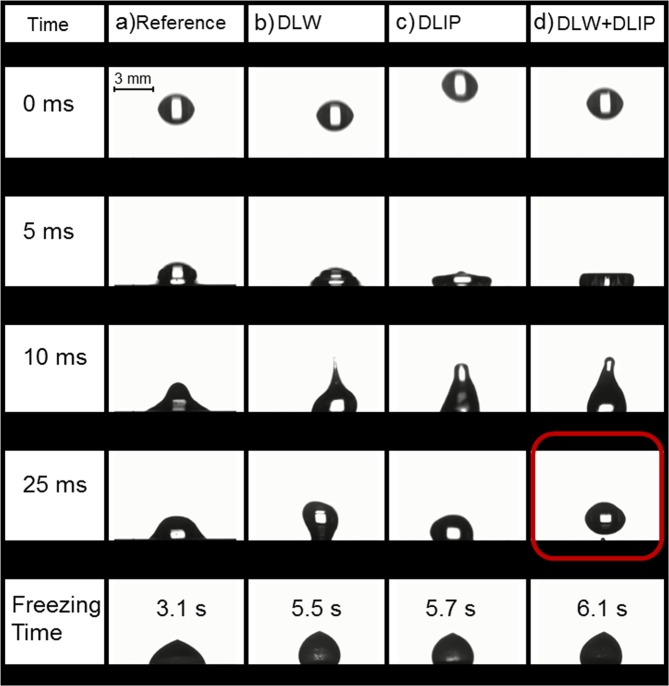
Images of the impinging scene of a 13 µl droplet after 0, 5, 10, 25 ms and after 10 to 30 s on an untreated reference (**a**), a DLW structure (**b**), a DLIP structure (**c**) and a DLW + DLIP structure (**d**). The red box on the DLW + DLIP structure points to the completely repelling droplet after 25 ms (see Supporting Videos 1–4).

Figure 8 shows selected snapshots of the impact dynamics on the studied structures. On the untreated reference (Fig. 8a), after the droplet was released (0 ms) it spread upon impact by the influence of gravitational force (5 ms). When the droplet retracts, its shape transformed from a cone-like (10 ms) to a truncated cone-like appearance (25 ms) and finally froze in a hemisphere-like state after 3.1 s. The droplets impinging on the DLW (Fig. 8b), DLIP (Fig. 8c), and DLW + DLIP (Fig. 8d) structured surfaces showed a larger spread upon impact (5 ms) compared to the reference, which lead to an elongated shaped during the retraction stage (10 ms). Remarkably, only the droplet impinging on the hierarchically patterned sample bounced off the surface (see red box in Fig. 8d**)**, while the droplets on the other structured surfaces remained wobbling in a more round-like shape on the surface (25 ms).

Finally, the droplets on the DLW, DLIP and DLW + DLIP patterns froze after 5.5 s, 5.7 s and 6.1 s, respectively. The difference between the freezing times shown in Fig. 8 compared to the results depicted in section 2.4 is related to the dynamic influence of this experiment, which was not mainly studied in this work^62,63^. Therefore, it can be reasonably assumed that the increased freezing time in the hierarchical texture is a direct consequence of the droplet rebounding. It is suspected that this behaviour is influenced by the hierarchically based superhydrophobicity^64–68^, suggesting that this complex structure is a promising approach for further investigations in the field of dynamic water and ice repellent surfaces.

Regarding the practical application of the DLW and DLIP techniques for producing hierarchical surface patterns, the processing time for 1 cm² calculated using the parameters in this work for the DLW and DLIP technologies are 240 s and 506 s, respectively. However, the used processing conditions were not optimized for high-throughput manufacturing. For instance, using a laser power of 240 W at 240 KHz, the DLW process can be optimized by a factor of 8 using only 2 m/s scanning speed, being capable to process 1 cm² in 30 s. Furthermore, the processing time for 1 cm² can be further decreased to only 6 s when using 1.2 kW of laser power and a scanner with 10 m/s linear speed. In the case of DLIP, processing speeds up to ~0.9 m²/min have been already reached, corresponding to 0.007 s for treating 1 cm². Furthermore, efforts for further improving the throughput for the DLIP process are being performed, with the objective of reaching up to 5 m²/min using a 1.5 kW IR ps-laser source combined with a polygon scanner^69^. Also, a concept for combining both methods has already been recently demonstrated^70^.

## Summary and Conclusions

In this work, triangular-like, pillar-like and hierarchical micro-structures were patterned on pure aluminium Al2024 surfaces using direct laser writing, direct laser interference patterning and the combination of both methods. The wetting properties of the fabricated structures were investigated over a 95 days period at a temperature of 20 °C. All three produced geometries showed a superhydrophobic with WCA higher than 150° after 12 to 15 days.

In addition, it was found that the wetting behaviour depends strongly on the sample temperature and it can be roughly divided into three regimes over a temperature range from −30 °C to 80 °C. In the range between 20 °C and 80 °C no significant variations of the contact angle was observed at different substrate temperatures resulting in slightly varying WCAs around the level of superhydrophobicity (150°). In the range between −20 °C and 20 °C, a linear decrease of the WCA with decreasing temperature for all examined surfaces was measured, which was attributed to condensation of moisture and frost formation. In the third range between −30 °C and −20 °C, the WCA increased with decreasing temperature, which was explained by the rapid freezing of the droplet at these extremely low temperatures.

The anti-icing characterisation revealed that in all laser-structured surfaces the freezing time of the 8 µl droplets increased up to three times compared to the unstructured reference surface. These findings should potentially result in a significant delay of ice formation in real environment applications. Heat transfer simulations based on the finite element method suggested that the reason for the observed freezing delay can be attributed to larger contact angles of the structured samples, leading to a smaller contact area between the surface and the droplets and also to higher droplets with increased internal thermal resistance. From these simulations, it was also found that the increase in the thermal resistance caused by the trapped air in the structures is responsible for an increase of the freezing time of only 6% and is therefore not mainly responsible for the delayed ice formation.

Droplet impingement tests showed that only on the hierarchical patterned Al samples produced by DLW and DLIP the droplets did not stick immediately on the surface, but bounced off the sample. Further investigations are still needed to shed light on the dynamics of droplets impinging on hierarchical laser structured surfaces and its potential for technical applications. The DLIP and DLW + DLIP microstructures could be therefore better suited for self-cleaning applications in the future.

## Materials and Methods

For the structuring experiments, 2 mm thick pure aluminium (Al 2024) sheets were cut into 50 mm × 50 mm pieces. Then, the samples were polished obtaining a surface roughness S_q_ of 41.5 nm ± 10 nm. Prior to the laser processing, the samples were cleaned from contamination using isopropanol in an ultrasonic bath for 10 minutes. After the laser process, the structured samples were stored under atmospheric conditions and any additional treatment was performed^43^.

**Figure 9:**
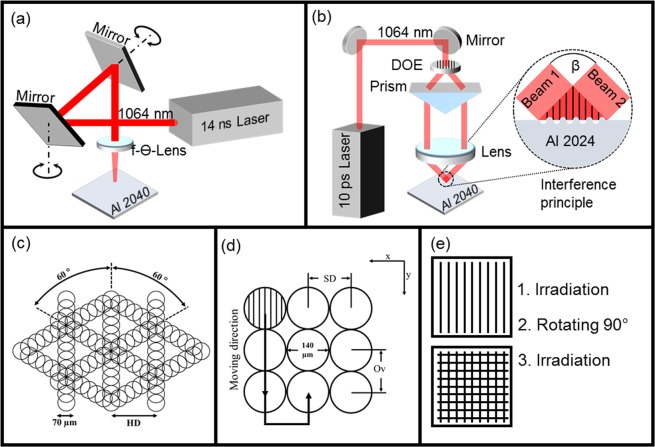
Schematic drawing showing the optical elements of the used DLW setup (**a**) and DLIP setup zooming to the interference process on the sample (**b**). Processing strategy using the DLW method (each circle represents a single laser pulse with a beam diameter of ~70 μm; HD – Hatch distance) (**d**). Processing strategy used for the DLIP method (SD – separation distance; Ov – Overlap); (**e**) strategy used for producing the pillar structures^43^.

The samples were processed using a laser surface texturing workstation (GF machining solutions P 600), which implements DLW technology (Fig. 9a). The system is equipped with a galvanometer scanner system including two mirrors and an IR (1064 nm) Ytterbium fibre laser with a maximal output power of 30 W. The pulse duration can be adjusted from 4 ns up to 200 ns. In our experiments, 14 ns pulse durations were used and the repetition rate was set to 30 kHz. The beam was focused onto the sample using a 254 mm focal length F-theta objective, obtaining a spot with a diameter of 70 µm. The laser fluence was set to 1.06 J/cm² by controlling the laser power. The laser beam was scanned along the surface of the sample with a constant speed of 250 mm/s. In order to achieve a sufficient material removal, the structuring process was repeated 10 times in all cases. The triangular-like surface structures processed by this setup consist of 3 sequentially formed line-like structures, whose orientation was each time rotated by 60°. The distance between the parallel lines (hatch distance, HD) was fixed to 50 µm^43^. The used strategy for producing the triangular features is shown in Fig. 9c.

The samples were also processed using the direct laser interference patterning (DLIP) technology (Fig. 9b). The used configuration utilizes an IR (1064 nm) solid-state Nd:YVO_4_ laser (Edgewave PX200) with 10 ps pulses and maximum output power of 10 W. The workstation is equipped with a commercial available DLIP optical head (manufactured by Fraunhofer IWS, Germany), which uses a diffractive optical element (DOE) to split the initial laser beam into two sub-beams. These two beams are guided to a prism where they are parallelized and then focused on the sample surface using a 60 mm aspheric lens. Using this configuration, a line-like interference intensity profile is obtained within a circular area with a diameter of 140 μm (Fig. 9b). In this study the angle between the beams was set to 8.7° to obtain a spatial period of 7.0 μm. The laser fluence was set to 1.93 J/cm² and the repetition rate of the laser was fixed to 1 kHz. The samples were translated in x- and y-directions by using high-precision axes (Aerotech, USA) with an accuracy of ± 2.5 μm^43^. By moving the sample in the y-direction (see Fig. 9d), the pulse-to-pulse overlap (Ov) can be controlled, which finally determines the number of laser pulses hitting a certain position. Differently, the x-direction translation, called separation distance (SD), mainly determines the homogeneity of the surface texture^43^. In this study, Ov and SD were 99% and 141.6 µm, respectively. To obtain the pillar-like structures, it is necessary to rotate the samples by 90° and repeat the process (Fig. 9e).

For analysing the surface topography of the textured surfaces, a confocal microscope (Sensofar S Neox) with a 150× objective was used, resulting in vertical and lateral resolution of 2 nm and 140 nm, respectively. The substrates were also evaluated using a scanning electron microscope (ZEISS Supra 40VP) at an operating voltage of 5.0 kV.

The static water contact angle (WCA), contact angle hysteresis (Hys) and sliding angle (SA) measurements were performed using a contact angle system (Krüss DSA 100 S), which is equipped with a special temperature chamber (Krüss TC40) based on a Peltier-element that permits to vary the temperature of the samples from −30 °C to 160 °C^43^. The room temperature outside the chamber was 20 °C. The system permits to control the relative humidity using an air flow with a pressure of ~0.5 bar (50 kPa) inside the chamber. For the WCA, Hys and SA measurements, 8 μl droplets of deionized water were used. The static water contact angles were calculated using the Young - Laplace and the tangent drop profile fitting methods and each point is the average of five measurements^43^. In all experiments, the droplets were positioned automatically on the surface. For the ice-repellence test, the freezing process was monitored and recorded by a CCD camera with a frame rate of 50 fps. To record the dynamic impact of the water droplets on the textured and non-textured samples, the frame rate was set to 200 fps. In the last case, the droplets had a volume of 13 µl and fell perpendicularly to the substrates from a height of 20 mm.

The surface chemistry analysis of the DLW + DLIP treated sample was performed using X-Ray photoelectron spectroscopy (XPS) method with an Amicus spectrometer (Kratos Analytical, UK) equipped with a non-monochromatic Mg K_α_ X-ray source (operated at 240 W and 8 kV). The kinetic energy of the photoelectrons was determined using an analyser with a pass energy of 75 eV. XPS analyses of the DLW and DLIP structures have been already reported in a previous work and thus were not included in this study^43^.

The heat transfer between the cold surface, the trapped air in the patterned cavities, the water droplet and the ambient air was modelled with the finite element method using the commercial software COMSOL Multiphysics 5.3. Considering that the droplet and air cavities are small enough and that the temperature gradient has the opposite direction to buoyancy, convection inside the droplet and within the trapped air is disregarded. Heat radiation is neglected as well due to the relatively small temperature gradients between the simulation domains. Therefore, the droplet freezing dynamics is reduced to a one-phase Stefan’s problem^60,71^ and is solved by finding the solution of the time-dependent heat balance equation together with Fourier’s heat conduction equation. The phase change occurring inside the droplet is simulated according to the apparent heat capacity formulation^72,73^. For simplicity, the volume expansion during the solidification of water is not considered, since the differences in the density of ice and water are lower than 8%^61,74^. Therefore, the density of water in liquid and solid state was assumed to have a constant value of 1000 kg m−^3^. To keep the computational time short and memory requirements low, the simulation domain was modelled assuming axial symmetry around the droplet vertical axis. The shape of experimental droplets with varying water contact angle was reproduced assuming an oblate ellipsoid cap shape and adjusting for each measured droplet the ellipsoid semi-axes and the diameter of the circular region at the droplet/sample surface interface. The temperature of the substrate is held constant at −20 °C, whereas the initial droplet temperature is set at 20 °C. The substrate surface patterns are sine-like textures with varying period of 7 µm, 20 µm and 50 µm, and structure heights of 5 µm, 15 µm and 35 µm, respectively. The inward heat transfer from the ambient to the droplet is modelled by the natural convective boundary condition given by

3 $$q=h\cdot (T-{T}_{ext})$$

where *q* is the inward heat flux normal to the boundary, *T* is the temperature on the boundary, and *T*_ext_ = 20 °C is the external ambient temperature. The temperature-dependent heat transfer coefficient *h* corresponds to the heat transfer on a sphere under natural convection conditions with air as fluid according to the approach already proposed^75^. The characteristic sphere radius is set to 1.24 mm, which is the radius of a sphere with a volume of 8 µl, as the experimental droplets. The temperature-dependent thermal conductivity and specific heat of water, ice and air, and the water latent heat of fusion were taken from the COMSOL built-in materials library. The simulation time step is set to 20 ms and the mesh size ranges from 15.000 to 80.000 elements, depending on the modelled surface and contact angle. The droplet freezing time is defined as the time when all the droplet volume is transformed into ice.

## Data Availability

The datasets generated and analysed during the current study are available from the corresponding author on reasonable request.

## Supplementary Video

Video 1
Video 2
Video 3
Video 4
